# Intercropping for Management of Insect Pests of Castor, *Ricinus communis*, in the Semi—Arid Tropics of India

**DOI:** 10.1673/031.012.1401

**Published:** 2012-02-01

**Authors:** M. Srinivasa Rao, C.A. Rama Rao, K. Srinivas, G. Pratibha, S.M. Vidya Sekhar, G. Sree Vani, B. Venkateswarlu

**Affiliations:** Central Research Institute for Dryland Agriculture (CRIDA), Hyderabad, India

**Keywords:** cultural practices, ecosystem diversity, natural enemies, agronomical evaluation, gross margin

## Abstract

Intercropping is one of the important cultural practices in pest management and is based on the principle of reducing insect pests by increasing the diversity of an ecosystem. On—farm experiments were conducted in villages of semi—arid tropical (SAT) India to identify the appropriate combination of castor (*Ricinus communis* L.) (Malpighiales: Euphorbiaceae) and intercropping in relation to pest incidence. The diversity created by introducing cluster bean, cowpea, black gram, or groundnut as intercrops in castor (1:2 ratio proportions) resulted in reduction of incidence of insect pests, namely semilooper (*Achaea janata* L.), leaf hopper (*Empoasca flavescens* Fabricius), and shoot and capsule borer (*Conogethes punctiferalis* Guenee). A buildup of natural enemies (*Microplitis*, coccinellids, and spiders) of the major pests of castor was also observed in these intercropping systems and resulted in the reduction of insect pests. Further, these systems were more efficient agronomically and economically, and were thus more profitable than a castor monocrop.

## Introduction

Castor (*Ricinus communis* L.) (Malpighiales: Euphorbiaceae) is an important non—edible oilseed cash crop grown across many parts of arid and semi—arid regions in India. Castor is grown for its beans, which yield non—edible oil mainly used in the manufacturing of paints, lubricants, soaps, hydraulic brake fluids, polymers, and perfumery products, among others; there are several derivatives of castor oil that are used in a variety of industries. Fluctuations in productivity levels of castor are experienced for two main reasons: first, it is grown as a rain—fed crop, and as a long—duration crop is subject to the vagaries of the monsoon; second, it attracts a large number of pests including the semilooper, *Achaea janata* L. (Noctuidae: Lepidoptera), and the shoot and capsule borer, *Conogethes punctiferalis* Guenee (Crambidae: Lepidoptera). *Achaea janata* and *C. punctiferalis* occur during early and late stages of crop growth, respectively (Singh 1987). Incidence of *A. janata* is generally noticed from vegetative to early reproductive phase of the crop (Tahiliani 1985; Basappa and Lingappa 2001). A peak level of infestation of *A. janata* causes excessive defoliation affecting photosynthesis. Later, the larvae eat away the tender capsules of primary and secondary spikes. It is estimated that castor yields are reduced by 30–50% due to *A. janata* alone. Incidence of *C. punctiferalis* is commonly noticed in the later stage of crop growth, especially secondary and tertiary spike orders. Larvae web the tender capsules, bore into them, and eat away the kernel. The borer attacks various plant parts such as the shoots, inflorescences, and capsules, causing considerable yield losses (Singh et al. 1992).

Intercropping is an important cultural practice in pest management and is based on the principle of reducing insect pests by increasing the diversity of an ecosystem (Risch 2005). Several studies indicate that diversification practices such as intercropping in pigeon pea and other crops are beneficial because these practices reduce pest damage (Srinivasa Rao et al. 2004; Songa et al. 2007). In castor, excessive branching allows the plant to have a unique plant structure, and offers an opportunity for manipulating the environment with different intercrops for possible reduction in incidence of insect pests. Farmers in the rain—fed regions, with their limited investment capacity, cannot afford input—intensive plant protection measures. Adoption of intercropping methods offers an opportunity to protect the crops by natural pest management. There is also a strong need to develop pest management practices that are affordable for resource—poor farmers. Thus, there is a considerable need to develop a system that is diverse and less prone to pests and diseases. When other pest management technologies are superimposed on such systems, it becomes much easier and cheaper for farmers to manage pests rather than in monocultures, which are more prone to pest incidence and require considerable investments in pest management. With these considerations in view, this study attempted to examine how the incidence of insect pests differs in an intercropping system compared to a castor monocrop.

## Materials and Methods

Experiments were conducted in farmers' fields in two villages of Rangareddy district in the state of Andhra Pradesh, India. The district is situated at the heart of Deccan plateau of the Indian subcontinent and lies between 16° 19′ and 18° 20′ N latitude and 17° 30′ E longitude, having a SAT (semi-arid tropical) climate with 31.68 °C mean annual temperature and 781.5 mm mean annual rainfall, which is representative of SAT India. In this region, cropping is limited to a single rain—fed crop per year, grown in the southwest monsoon season from June–September. The crops grown in this region are castor, maize, and pigeon pea with sorghum intercrop. After conducting participatory rural appraisal of the villages, five enthusiastic farmers in each village with similar fields (soil type, slope, and surrounding vegetation) were selected for conducting the experiments. The soils were shallow red sandy loams with low water holding capacity and poor soil fertility. The fields were located on nearly level land (< 2% slope). The selected farmers were given the objectives of the study and protocol to be followed. Guides were positioned in each of the villages to supervise the conduct of the experiments and to facilitate communication between the farmers and the research team. The experiment was laid out as a randomized complete block design (RCBD) with nine treatments (eight castor—based intercropping systems and one castor monocrop as control) and the ten farmers as replicates. In each field an area of 9000 m^2^ was demarcated for the experiment. The area was divided in to nine longitudinal strips (100 m × 10 m) with 2 m walkways on either side of the strips. The nine cropping systems were randomly allocated to the nine strips in each of the fields. All measurements on incidence of insect pests and occurrence of natural enemies and crop yield were recorded from these strips. The experimental fields were plowed and leveled before the crops were sown. Castor (*R. communis*) is most important non—edible oil seed crop grown in India during the June– December rainy season. Castor and intercrops were sown using a planter in furrows opened in the first two weeks of June. Castor rows were spaced 90 cm apart.

The rationale for the selection of intercrops to quantify the impact of intercrops on pest incidence is as follows. Black gram and green gram are short—duration legumes grown by farmers. Cowpea is a short—duration pulse crop also considered as an eco—feast crop, which attracts aphids, thus increasing occurrence of coccinellids (Surulivelu 2004) and encouraging multiplication of coccinellids and other predators. Pigeon pea is a long— duration crop grown by farmers along with castor. Cluster bean is considered a relatively insect pest—free crop and was found to record higher LER (land equivalent ratio) values when intercropped with castor (Subba Reddy et al. 2004). Groundnut and sunflower are grown as intercrops in pigeon pea and castor by some farmers. Sorghum is the major food cereal crop grown in dryland conditions. Intercropping systems that have been found to be effective along with some other systems, both popular and/or suggested by the farmers were included.

Intercrops were sown in between rows of castor in an additive manner to keep the population of castor plants constant across the nine cropping systems. All other intercrops were sown in two rows 30 cm apart from each other and 30 cm away from castor rows on either side. Routine agronomic practices such as application of recommended doses of fertilizers to castor, intercrops, and interculture were taken up at appropriate growth stages of the crops. No pest control measures were undertaken during the entire crop growth period.

Weekly insect counts were recorded from 10 randomly labeled castor plants in each plot at weekly intervals. Sampling and recording of the population was done from the different plants of the respective plots at weekly intervals. Three terminals per plant were selected for recording the absolute population. Field observations of insect pest and predator populations were recorded during the cool hours of the day (07:00–09:30 and 16:00– 18:00) (Pradhan 1964). The abundance of insect pests and predators was determined for each system at weekly intervals in 2003 and 2004. Crops were harvested at different intervals from first order spikes (primaries), second order spikes (secondaries), third order spikes (tertiaries), and so on. Capsules were obtained from the respective spike orders. The data recorded on damage caused by *C. punctiferalis* across intercropping systems was collected individually by spike order (at the time of harvest of each primary, secondary, and tertiary); thus, percent damage was also estimated. Various species of coccinellid predators were observed, namely *Menochilus sexmaculatus, Brumoides suturalis, Illois indica, Coccinella transversalis*, and *C. septempunctata. Menochilus sexmaculatus* was found to be most dominant, accounting for more than 80% of the total coccinellid population. The coccinellids were considered a group, and their presence was recorded in all the intercropping systems. Four species of spiders were observed in the intercropping systems under study. These belong to the families Clubionidae, Araneidae, Linyphilidae, and Thomisidae. Among various spiders recorded, *Clubiona* spp. was dominant. All the spiders, irrespective of the family to which they belonged, were recorded together as one unit. The occurrence of endo— parasitoid *Microplitis* spp. on *A. janata* was monitored at weekly intervals, neonate larvae were collected and reared in the laboratory, formation of cocoon at the posterior region of the larvae were recorded, and adult emergence of braconids was represented as percent parasitism. Mean insect numbers and percent damage and natural enemy counts across sampling intervals were determined to provide a single index of pest/natural enemy population for making comparisons across intercropping systems. The final pooled mean data was analyzed and presented. Seed yields of castor and intercrops were determined by harvesting a constant area of 60 m^2^. The seed yield of intercrops was converted into castor equivalent yield on the basis of farm harvest prices of the crops (Bureau of Economics and Statistics 2005). Land equivalent ratio (LER) for the systems were worked out as suggested by Willey and Rao (1980). Intercropping system effects were analyzed using one—way ANOVA. Cropping systems means were compared and separated by least significant difference at *p* < 0.05 using SPSS version 11.0. Focus group discussions were held with the 10 participating farmers and 15 other neighboring farmers at the end of experiments. Farmers were asked to rate each intercropping system on a scale of 1 to 10 with respect to the three parameters (pest incidence, yield, and cost of cultivation).

## Results

### Impact of intercropping on insect pests

In castor—based intercropping systems, the incidence of *A. janata, E. flavescens*, and *T. ricini* were predominantly noticed; mean population per plant and mean percent damage by *C. punctiferalis* are mentioned in Table 1. The incidence of *A. janata* was uni— modal, with peak infestation noticed only once when it coincided with the formation of primaries and the incidence varied across the intercropping systems. The intercropping systems castor + cluster bean, castor + cowpea, castor + groundnut, and castor + pigeon pea recorded significantly lower population levels (0.58 to 0.89 per plant) (*F*_8,32_ = 30.27, *p* < 0.01). The higher level of *A. janata* population was observed in castor + green gram, castor + black gram, and castor monocrop (1.12 to 1.29 per plant). The significant effect of these intercropping systems on the population of *C. punctiferalis* was observed. The intercrops significantly (*F*_8,32_ = 8.26, *p* < 0.05) altered capsule damage, and were less with cluster bean (9.89%), black gram, green gram, cowpea, and groundnut (10.35 to 13.38%), and were significantly superior over the rest of the intercropping systems and castor monocrop (20.84%). The population of *E. flavescens* and *T. ricini* were also fluctuated significantly and varied across different intercropping systems during the crop period. Significant reduction of incidence of these pests was not observed over monocrop of castor (*F*_8,32_ = 1.27, *p* > 0.05).

### Impact of intercropping on natural enemies

**Figure 1.**
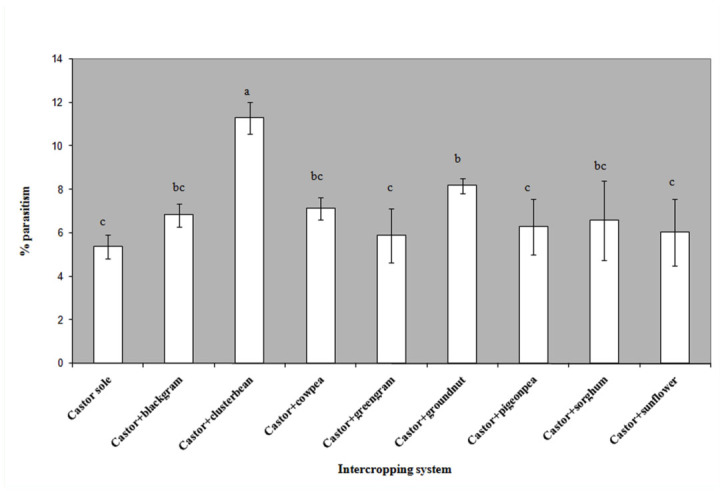
Mean occurrence of *Microplitis* on *Achaea janata* across various intercropping systems. High quality figures are available online.

Diversity in within crop canopy and poly crop situations often reduced the incidence of insect pests (Risch et al. 1983). This was mainly due to *in—situ* culturing of natural enemies well—within crop ecosystems, and the same hypothesis was tested in castor crop. The presence of *Microplitis* cocoons at the posterior end of larvae and adult emergence were recorded and noted as indicators of parasitism. The percent parasitism varied across intercropping systems between 5–11%. A higher level of parasitoid attacks on *A. janata* was recorded in castor + cluster bean (11.29 ± 0.72%) followed by castor + ground nut (8.17 ± 0.34%) which were significantly (*F*_8,32_ = 9.12, *p* < 0.05) higher than the rest of the systems. Parasitoid activity was higher on castor crops with cowpea, black gram, and sorghum as intercrops (Figure 1). The activity of coccinellids was recorded within a month after sowing and continued through the entire crop growth, and during the later lower levels of coccinellid populations. The peak activity of coccinellids was recorded during the formation of primaries in all intercropping systems. The coccinellid population varied significantly across intercropping systems throughout the crop growth period. Systems like castor + cluster bean, castor + cowpea, castor + black gram, castor + green gram, and castor + groundnut had significantly higher populations of coccinellids than the other intercropping systems, that was reflected in mean number (0.38 to 0.48 per plant) compared to the castor monocrop (0.13 per plant) (*F*_8,32_ = 6.27, *p* < 0.01). The fluctuation of the spider population was significant among intercropping systems, and the spider activity was significantly higher in castor + cluster bean (0.41 per plant). The presence of parasitoids such as *Telenomous* sp. and *Trichogramma* sp. was noticed on eggs of *A. janata.* The level of parasitism was sparse (< 5%). The larval parasitoid *Euplectrus* sp. is a gregarious ecto—parasitoid that attacks semilooper larvae, and nearly 5–10 individual *Euplectrus* could feed on single larva of *A. janata.*

**Table 1.**
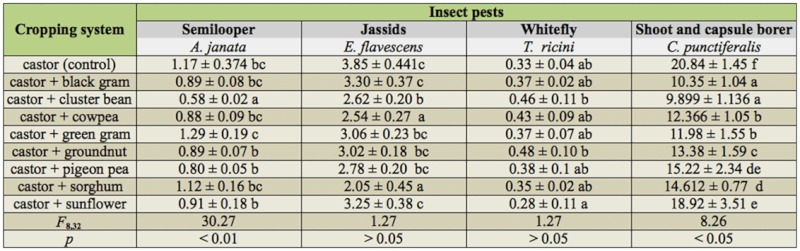
Mean number of insect pests per plant ± SD in castor based intercropping systems.

**Table 2.**
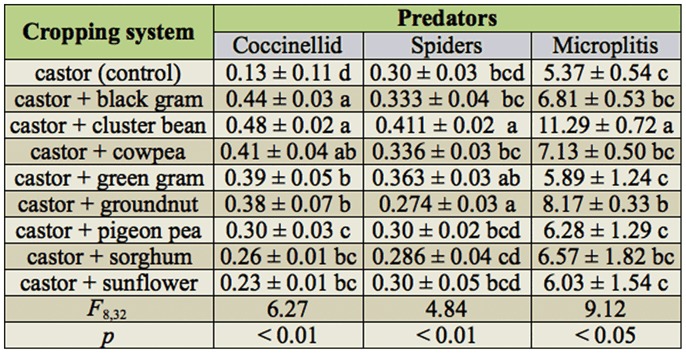
Mean number of predators per plant ± SD in castor based intercropping systems.

### Castor equivalent yields and LER

**Figure 2.**
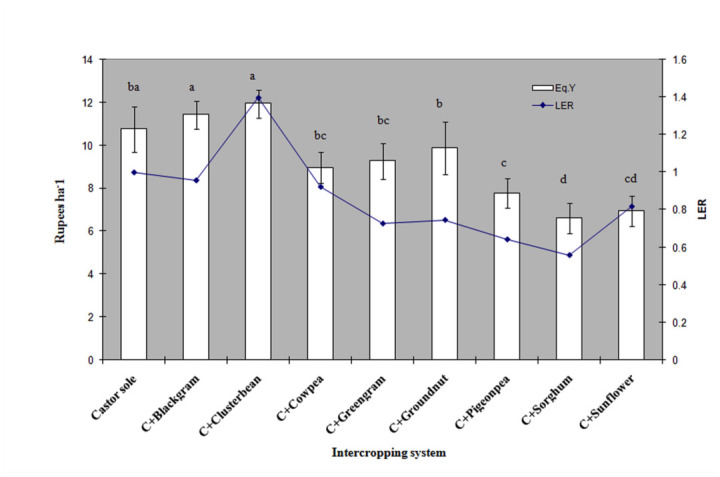
Impact of intercropping on castor equivalent yields and land equivalent ration (LER). High quality figures are available online.

Equivalent yields of castor were significantly higher in castor with cluster bean (11.93 ± 0.65 qha^-1^) followed by black gram (11.42 ± 0.64 qha^-1^) as intercrops (Figure 2). Lower equivalent yields were recorded in castor + sorghum (6.60 ± 0.69 qha^-1^), castor + sunflower (6.93 ± 0.71 qha^-1^) and castor + pigeon pea (7.70 ± 0.70 qha^-1^) compared to a castor monocrop. The intercropping systems were observed with respect to land utilization; land equivalent ratios are presented in Figure 2. The LER values were highest in castor + cluster bean (1.39) followed by castor + black gram and castor + cowpea (i.e., more than one). All remaining systems recorded lower LER values than castor alone. The lowest LER was observed in castor with sorghum (0.56) followed by pigeon pea (0.64) and green gram (0.73) as intercrops.

### Economics

**Figure 3.**
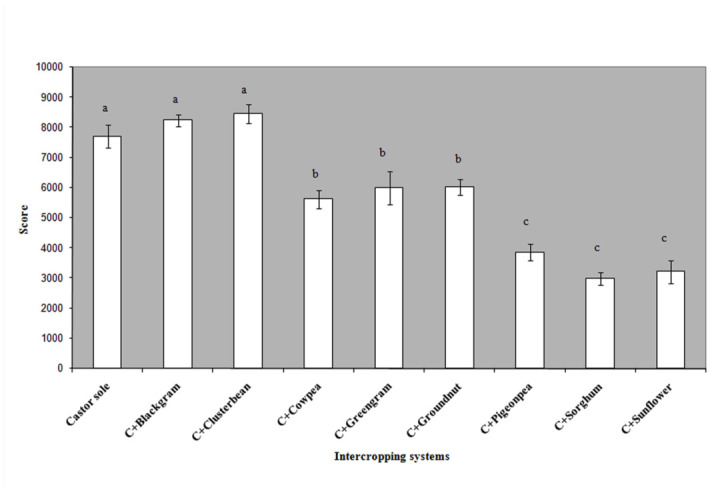
Impact of intercropping on gross margin from castor. High quality figures are available online.

The economics of castor—based intercropping systems revealed that the gross margin, as indicated by returns over variable costs, was found to be highest in the case of castor + cluster bean (8463 ± 315 Rsha^-1^) (1 USD = 40 rupees) and castor + black gram (8234 ± 203 Rsha^-1^) (*F*_8,32_ = 164.72, *p* < 0.01). The Systems castor + sorghum (2995 ± 207 Rsha ^-1^) and castor + sunflower (3212 ± 378 Rsha^-1^) recorded less returns (Figure 3).

### Farmer feedback

Focus group discussions were held with farmers before commencement of the on—farm experiments. The discussions revealed a number of important pieces of information: farmers were routinely practicing castor monocropping; few farmers were aware of the advantages of intercropping in terms of lowering of pest infestation; adoption of chemical insecticides was the common solution for controlling insect pests; and although some farmers were aware of the various components of IPM, implementation was not significant.

**Figure 4.**
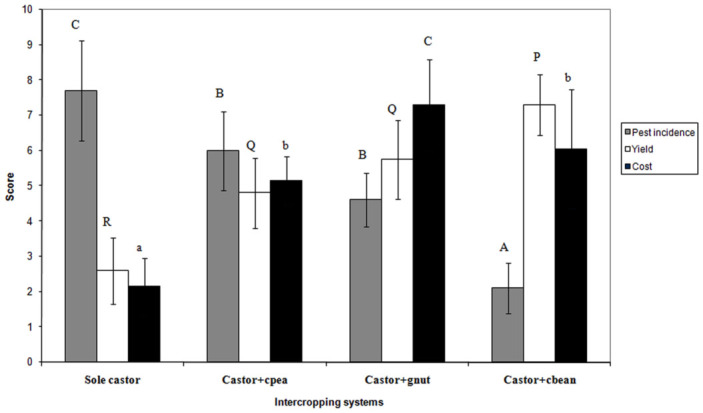
Rating by farmers of different castor–based intercropping systems. High quality figures are available online.

Similar focus group discussions were held again at the end of the project with the same set of farmers as before. Farmers were asked to rate each intercropping system on a scale of 1 to 10 with respect to three parameters, pest incidence, yield, and cost of cultivation. Farmers expressed that the diverse crop systems in fact experienced lower pest infestation than the monocultures. The obtained average indicated that the castor + cluster bean system fared better with a score of 2.1 ± 0.72 for pest incidence, 7.3 ± 0.86 for yield, and 6.05 ± 1.69 for cost and was considered superior to all other systems (Figure 4).

However, the costs incurred were least with a castor monocrop (2.15 ± 0.81). Farmers also thought that the yield from the castor + groundnut and castor + cowpea systems were comparable to that of castor + cluster bean, but required higher investments towards seed and picking charges.

As a result of frequent visits by project staff, farmers developed the ability to recognize a number of insect pests, their feeding habits, and natural enemies. The benefits of choosing an intercrop (higher yields, saving on cost, and better cash flow) were also better appreciated by the farmers.

## Discussion

The present results showed that intercropping had positive influence with cluster bean, cowpea, groundnut, and black gram as intercrops, which reduced infestation. Deterrence of pest colonization through intra— field diversity is probably one of the more promising means of controlling insect pests. Diversity in the crop field may have a profound effect on colonization by insects, and has been well—documented in the case of intercropping (Risch et al. 1983). Any such delay in pest colonization results in subsequent delays in the pest buildup; this was observed with intercrops like cluster bean, cowpea, black gram, and groundnut. The present results showed that intercropping had positive influence with dicrop combination, which reduced infestation. This suggests that pest migration after initial establishment was possibly inhibited by the non—host plants as physical barriers to inter— or intra—row migration within intercrop treatment, which had both sorghum or groundnut arranged in rows. The conspicuous pest reduction in cluster bean—castor dicrop combination may be because cluster bean made inter—row migration very difficult for *A. janata* and *C. punctiferalis* during crop growth, especially in the later stages of castor crop development as reflected in reduction of *C. punctiferalis* damage. The intercrops facilitated the natural proliferation of predators and recorded higher populations of coccinellids and spiders. Srinivasa Rao et al. (2004) reported the increased activity of coccinellids and spiders in leguminous intercrops. The low incidence of insect pests in intercrop systems was often attributed to one factor (i.e., higher abundance) of their parasitoids and predators, which supports the “natural enemies hypothesis”. Aphids in pulses (cowpea and black gram) and whitefly in cluster bean represented the main prey, which in turn attracted these generalist predators. Similarly higher populations of these predators were recorded in another crop like cotton. These predators on cowpea and cluster bean might have exercised a regulatory effect on pests of castor. Baliddawa (1985) observed that up to 30% of pest reduction in intercropping systems could be due to the “natural enemy effect”. The greater effectiveness of cluster bean and cowpea in reducing the insect pests on castor can be attributed to the combined operation of barrier and natural enemy effects. Many herbivores, especially those with narrow host ranges (such as *A. janata*), are most likely to find and remain on host plants that are concentrated, i.e., that occur in large dense or pure stands, which constitutes the principle of the “resource concentration hypothesis”.

Associational resistance refers to the reduced herbivore attack that a plant experiences when they are associated with taxonomically different plants (Kaitaniemi et al. 2007), and it may be true for the intercropping systems. Associational resistance is due to either resource concentration, the natural enemy hypothesis, or both. Favorable intercrops like cowpea and cluster bean recorded higher populations of predators and facilitated *in—situ* culturing of them. The castor + sorghum intercropping system had lower pest populations. Although it is not a legume, sorghum as an intercrop reduced the pest incidence in castor. Because of its faster growth and canopy formation, the sorghum crop suppressed growth of the castor plants and made them small and lean. Plants suppressed in this way may become less attractive to a pest or provide a less suitable food source; alternatively, the smaller suppressed plants may constitute a less efficient crop for passively dispersing pests (Trenbath 1993).

## Conclusions

The diversity created by introducing cluster bean, cowpea, black gram, or groundnut as intercrops in castor resulted in a buildup of natural enemies (*Microplitis*, coccinellids, and spiders) of the major pests of castor, also resulted in less congenial conditions for insect pests such as *A. janata* and *C. punctiferalis.* As a result of the buildup of natural enemies, there was much less pest incidence and damage in castor intercropped with cluster bean, cowpea, and groundnut compared to the castor monocrop. Further, these systems were more efficient agronomically in terms of equivalent yields and land equivalent ratio. Economic analysis also showed that these intercropping systems were more profitable than castor alone. It can be concluded that these systems are better protected from pest attacks, resulting in higher yields and economic returns.
